# Distinct contributions of MSL complex subunits to the transcriptional enhancement responsible for dosage compensation in *Drosophila*

**DOI:** 10.1093/nar/gks890

**Published:** 2012-10-09

**Authors:** David Dunlap, Ruth Yokoyama, Huiping Ling, He-Ying Sun, Kerry McGill, Simona Cugusi, John C. Lucchesi

**Affiliations:** ^1^Department of Cell Biology and ^2^Department of Biology, Emory University, Atlanta, GA 30322, USA

## Abstract

The regulatory mechanism of dosage compensation is the paramount example of epigenetic regulation at the chromosomal level. In *Drosophila*, this mechanism, designed to compensate for the difference in the dosage of X-linked genes between the sexes, depends on the MSL complex that enhances the transcription of the single dose of these genes in males. We have investigated the function of various subunits of the complex in mediating dosage compensation. Our results confirm that the highly enriched specific acetylation of histone H4 at lysine 16 of compensated genes by the histone acetyl transferase subunit MOF induces a more disorganized state of their chromatin. We have determined that the association of the MSL complex reduces the level of negative supercoiling of the deoxyribonucleic acid of compensated genes, and we have defined the role that the other subunits of the complex play in this topological modification. Lastly, we have analyzed the potential contribution of ISWI-containing remodeling complexes to the architecture of compensated chromatin, and we suggest a role for this remodeling factor in dosage compensation.

## INTRODUCTION

Dosage compensation is a mechanism that renders the products of most X-linked genes equal in males and females. In *Drosophila*, the male specific lethal (MSL) complex mediates dosage compensation by enhancing the transcriptional rate of most X-linked genes in males [see reviews (1–3)]. The MSL complex associates with numerous sites on the X chromosome in somatic cells of males but not of females where it is prevented from forming by the presence of the Sex lethal (SXL) female-determining protein. In *Drosophila* males, the complex is believed to assemble at the locus of the two roX genes and then spread to numerous additional sites along the X chromosome for which it has a complete range of affinity levels (4,5). The MSL complex consists of a core of five protein subunits [encoded by male-specific lethal 1, 2 and 3 (*msl1*, *msl2* and *msl3*) and males absent on the first (*mof*) and maleless (*mle*)], as well as one of two noncoding ribonucleic acids (RNAs) [RNA on the X1 and 2 (*roX1* and *roX2*)]. MSL2 is an E3 ubiquitin ligase that ubiquitinates histone H2B at lysine 34 *in vitro* (6); its possible effects *in vivo* on dosage compensation are not easily determined because of MSL2’s role in assembly and targeting of the complex. MOF is a member of the MYST subfamily of histone acetyltransferases; it specifically acetylates histone H4 at lysine 16 (7). Amino acids 16–20 of the histone H4 tail constitute a basic segment that is thought to associate with an acidic patch formed by an H2A/H2B dimer on the surface of a neighboring nucleosome, contributing to chromatin condensation (8,9). Acetylation of H4 at K16 would reduce this effect. MLE is an adenosine triphosphate (ATP)-dependent, DEXH-box RNA/deoxyribonucleic acid (DNA) helicase. It unwinds short (<40 bp) double-stranded RNA or RNA/DNA duplexes with 3′ overhangs *in vitro* (10). It is related to the ATPases present in complexes that remodel chromatin by altering the positioning or the architecture of nucleosomes (11). Some of these ATPases have been shown to generate superhelical torsion in DNA or reconstituted chromatin fibers (12). In addition, ATP-dependent remodeling complexes, as well as in some cases their recombinant ATPase subunits, are able to disrupt nucleosome–DNA interactions by translocating DNA (13,14).

In multicellular eukaryotes the process of transcription consists of several distinct steps: the formation and activation of the initiation complex, elongation and termination. In numerous instances, the transcription complex pauses shortly after initiation and must be modified to engage in productive elongation. Although the processivity of the elongation complex, the ability of RNAPII (RNA polymerase II) to travel to the end of the gene without stopping or prematurely releasing the template, has drawn substantial attention, the rate of elongation has usually been assumed to be constant and, therefore, has been the subject of relatively few investigations. However, in *Drosophila* males, the high degree of acetylation of histone H4 at lysine 16 (H4K16ac) achieved by the MOF subunit of the MSL complex on dosage-compensated genes occurs throughout transcriptional units and actually tends to increase toward the 3′ end (15,16). Compensated genes exhibit an approximate 2-fold enhancement in levels of product irrespective of the strength of their promoters. These facts have led us to propose that dosage compensation is achieved by increasing the rate of elongation of the transcribing polymerases (16), a hypothesis that has been endorsed by others (17,18) and was recently supported experimentally (19). Naturally, to result in a 2-fold increase in the steady-state level of transcripts, an enhanced rate of elongation must be accompanied by an enhanced rate of reinitiation of transcription.

Several *in vitr*o experiments have demonstrated that the presence of H4K16ac in nucleosomes causes arrays to resist condensation (20–22) or weakens the self-association of single nucleosome particles (23). Using single chromatin fibers reconstituted with chemically synthesized H4K16ac, we have obtained evidence that the presence of H4K16ac is sufficient to disorder nucleosomal arrays, i.e. that this modification weakens chromatin fibers.

Recently we have established that the DNA of a plasmid that contains a sequence targeted by the MSL complex and a dosage-compensated transcriptional unit is less negatively supercoiled than the DNA of noncompensated transcriptionally active unit, and that this difference is not the consequence of the enhanced rate of transcription associated with dosage compensation (Cugusi *et al.*, submitted for publication). By means of RNA interference, we have determined that different subunits of the complex contribute incrementally to the difference in topology exhibited by compensated chromatin.

Finally, we have investigated the role of general chromatin remodeling complexes on the architecture of dosage-compensated templates. Partial loss-of-function mutations in ISWI (imitation switch protein), the ATPase common to four chromatin remodeling complexes: CHRAC (chromatin accessibility complex), ACF (assembly of core histones factor), NURF (nucleosome remodeling factor) and RSF (remodeling and spacing factor), transform the male X chromosome in salivary gland polytene chromosome preparations into an amorphous chromatin mass (24). X chromosome morphology can be rescued in males (or in females in which the formation of the complex has been induced and mutations in ISWI cause the same abnormal morphology of the X chromosomes as they do in males) by preventing the H4K16 acetylation. Our results suggest that ISWI may play a regulatory role in the level of expression of dosage-compensated genes to ensure that their level of expression is not enhanced beyond the required level.

All of these considerations provide a framework for the mechanistic basis of the transcriptional enhancement responsible for dosage compensation.

## MATERIALS AND METHODS

### Preparation of DNA

Plasmid pWM530_601-12X177 was provided by Timothy Richmond (25). This plasmid was digested with NgoMIV, AvaI and AvaII, and the ∼2200-bp fragment containing 12 repeats of the 601 sequence was isolated by gel electrophoresis, and ligated into the AvaI site of pWM530_601-12x177 to produce a clone (pWM530_601_24X177) with two tandem copies of the 12X177 601 repeats in the head-to-tail orientation.

To assemble the DNA for tethering microspheres, plasmid pWM530_601_24X177 was digested with SpeI and ApaLI to generate a 5049-bp fragment containing the 24 repeats and AvaII to digest the vector into small pieces. This DNA was differentially precipitated with polyethylene glycol to separate the repeat fragment from unwanted shorter fragments (26).

The repeat-containing fragment was ligated on one end to a digoxigenin-labeled DNA segment and on the other end to a biotin-labeled segment, each ∼1 kb in length, using a 3:1 ratio to produce an overall DNA tether ∼7 kb in length. The biotin-labeled DNA was produced by polymerase chain reaction (PCR) amplification using the forward-5′TGGGTGAGCAAAAACAGGAAGGCA and reverse-5′GCGTAATCTGCTGCTTGCAA primers on a pBluescript KS+ template (pBS, Stratagene) with 15% biotin-11-dUTP (Fermentas, Glen Burnie, MD, USA) substituting deoxythymidine triphosphate (dTTP) to produce a 1981-bp fragment. This was digested with XbaI to generate two ∼1-kb fragments, each with a complementary end for ligation to the SpeI overhang. The digoxigenin-labeled DNA was similarly produced using the forward-5′GCACTAAATCGGAACCCTAAAG and reverse-5′TTGAGTACTCACCAGTCACAG primers on pBluescript KS+ template with 15% digoxigenin-11-dUTP (Roche, Indianapolis, IN, USA) to produce a 2254-bp fragment. This was digested with ApaLI to generate ∼1.1-kb digoxigenin-labeled fragments for ligation.

### Preparation of histone octamers

Plasmids for expression of *Drosophila melanogaster* histones H2A, H2B, H3 and H4 were obtained from the laboratory of Dr Erica Mancini (Oxford University). Histones were prepared as previously described (27). Briefly, BL21 cells were transfected, and protein expression was induced with Isopropyl β-D-1-thiogalactopyranoside (IPTG). Cells were lysed, and proteins were purified from inclusion bodies as described. These were solubilized, washed and the proteins fractionated by gel filtration using a Sephacryl S200 column. Alternatively, recombinant *Xenopus laevis* histones were used (Cayman Chemical Company, Ann Arbor, MI, USA). Purified H2A, H2B, H3 and H4 were combined in unfolding buffer (6 M guanidinium chloride; 20 mM Tris–HCl, pH 7.5 and 5 mM DTT) in dialysis tubing with a 3.5 kD molecular cutoff (MWCO) and dialyzed into refolding buffer [2 M NaCl; 10 mM Tris–HCl, pH 7.5; 1 mM ethylenediaminetetraacetic acid (EDTA) and 5 mM 2-mercaptoethanol]. The reconstituted octamers were separated from aggregates, tetramers and dimers by gel filtration on a Superdex 200 column, and the fractions were analyzed by sodium dodecyl sulphate-polyacrylamide gel electrophoresis. Octamers were also reconstituted using *Xenopus* H2A, H2B, H3 and histone H4 acetylated at lysine 16 (obtained from Dr Lars Nordenskiöld, Nanyang University, Singapore).

### Reconstitution of chromatin and NuA4 acetylation of chromatin fibers

Purified octamers and ∼5 µg of DNA were mixed in 30 µl total volume at ratios of 0.8 to 1.3 octamer/601 binding site in Tris-HCl EDTA (TE) with 2 M NaCl and placed in Slide-A-Lyzer MINI Dialysis Devices, 10 K MWCO (Thermo Fisher Scientific, Rockford, IL, USA). The salt was lowered in steps to 1, 0.75 and finally 0.0025 M during the course of 2 days.

The Piccolo subcomplex of the NuA4 complex (6.5 µg/µl in 16 mM Tris–HCl, pH 8.0; 120 mM NaCl; 8 mM 2-mercaptoethanol; 0.08 mM EDTA and 20% glycerol) was provided by Dr Song Tan, Pennsylvania State University. Chromatin was reconstituted at a 1.3:1 ratio as described earlier and was incubated in 10% glycerol, 1X protease inhibitor cocktail (Thermo Fisher Scientific), 50 mM Tris–HCl, pH 7.5, with 3.25 µg of NuA4 and 2 µl (5.69 mmol/l) of Acetyl-CoA at 37°C for 30 min.

### Preparation of flow chambers

A detailed protocol is provided in the Supplementary Methods. Briefly, a serpentine channel was cut in a piece of parafilm and thermally adhered between No.1 50 × 22-mm coverglasses. The channel was rinsed with filtered phosphate buffered saline (PBS), and 50 µl of 20 mg/ml anti-digoxigenin (Roche) diluted in PBS was introduced into the chamber, incubated overnight at 4°C and flushed with two volumes of filtered PBS. A 1-µl aliquot of MyOne streptavidin-coated microspheres (Invitrogen, Carlsbad, CA, USA) was washed three times in 150 µl of 1 M NaCl in TE, resuspended in 150 µl of filtered PBS, introduced into the chamber and incubated for at least 10 min. This first aliquot of beads adhered directly to the surface to serve as reference beads. An additional 2 µl of MyOne beads was resuspended in 100 µl of 100 mM NaCl in TE. A volume of 2 µl (∼50 ng total DNA) of the ligation reaction was added to 100 µl of 100 mM NaCl in TE and mixed with the resuspended beads in a 1.5 ml microcentrifuge tube for 10 min, with gentle flicking every minute to prevent sedimentation of the beads. The bead–DNA solution was spiked with 4 µl of 0.1 mM d-biotin (Sigma, St Louis, MO, USA) in TE, mixed and incubated for 5 min. A volume of 50 µl of the bead–DNA solution was introduced into the microchamber. After 5 min, the microchamber was gently rinsed with three volumes of 100 mM NaCl with 0.2 mg/ml casein with 0.5% Tween in 20 mM Tris–HCl, pH 7.4, to remove untethered beads.

### Force extension measurements

A detailed description is provided in the Supplementary Methods. Tethered beads in the microchamber were observed in a custom-built magnetic tweezer implemented according to previous designs (28,29). N52 grade neodymium magnets (K&J Magnetics, Jamison, PA, USA) were placed above the microchamber straddling the optical axis to create up to a few tens of piconewton of tension at the minimum separation from the microchamber. For each tethered molecule exhibiting symmetric random motion, the tether length was recorded for a series of increments of tension from subpiconewton values up to ∼10 nN (Supplementary Figure S2). These data were plotted and fit to the expression for a worm-like chain with the contour and persistence lengths as variables (30).

### Atomic force microscope imaging

Reconstituted chromatin was diluted in 2.5 mM NaCl in TE to ∼1 nM concentrations of DNA. A volume of 5 µl of this solution was spotted on ruby muscovite mica (Ted Pella, Redding, CA, USA) that had been previously coated with poly-l-ornithine (31). After an incubation of 1–2 min, the surface was rinsed with high pressure (or high performance) liquid chromatography grade water and blown dry with a gentle stream of air from an aerosol duster.

### RNA interference (RNAi) knockdown

On the day before transfection, S2 cells were treated with 10 µg/ml double-stranded RNA (dsRNA). Additional dsRNA was added throughout the experiment to maintain the 10 µg/ml concentration. dsRNA was made following the Ambion MEGAscript protocol (Life Technologies, Grand Island, NY, USA) with primers as specified in the Supplementary Methods.

### Analysis of dosage compensation

Four days after transfection, S2 cells were collected, and luciferase activity was determined by using the dual luciferase reporter assay system (Promega, Madison, WI, USA). The firefly luciferase activity was normalized to *Renilla* luciferase activity for each sample. At least three independent experiments were performed; the results were averaged, and error bars in the figures represent standard deviations of the means.

### Analysis of topoisomers

Four days after transfection, total DNA was isolated and subjected to electrophoresis in 1% agarose gels containing 150 µg/ml of chloroquine. Following transfer to Hybond™-N^+^ membrane and Southern blotting with a DIG-labeled firefly luciferase gene probe (DIG High Prime DNA Labeling and Detection Starter Kit I, Roche), the topoisomer distributions were scanned using a Typhoon Trio densitometer (GE Healthcare Life Sciences, Piscataway, NJ, USA). The center of the topoisomer distribution was obtained by the method of Morse (32). Briefly, the band with the greatest intensity is assigned a linking number of zero and linking numbers of −1, −2, etc. or +1, +2, etc. are assigned to the slower and more rapid bands, respectively. A straight line with slope *m* and intersect *b* is obtained by plotting (1/Δ*Lk_i_*)_*_ln (*I_i_/I*_max_) versus Δ*Lk_i_*, in which Δ*Lk_i_* corresponds to the relative linking number assigned to a band *i* having relative intensity *I_i_*, and *I*_max_ is the intensity of the most intense band. The centers of the topoisomer distributions, indicating positive or negative changes in linking number, were ranked using the distance, *b/2 m*, from the most intense band.

### Nucleosome scanning assay

#### Transfection and MNase digestion

S2 cells were transfected with roX2 or Nesprin plasmids as previously described (33). The nucleosome positions on these plasmids were mapped following the protocol of Petesch and Lis (34). On the fourth day after transfection, the cells were cross-linked with a final concentration of 0.3% formaldehyde for 1 min. Following quenching by adding 125 mM glycine for 5 min at room temperature, nuclei were isolated at 4°C in hypertonic buffer (300 mM sucrose; 2 mM Mg acetate; 3 mM CaCl_2_; 10 mM Tris, pH 8; 0.1 M Triton X-100 and 0.5 mM DTT) using 20 strokes in a glass Dounce homogenizer. The nuclei were then centrifuged for 5 min at 720 g at 4°C. The nuclei were washed and centrifuged two more times in the same buffer. The nuclei were then resuspended in buffer D (25% glycerol; 5 mM Mg acetate; 50 mM Tris, pH 8; 0.1 mM EDTA and 5 mM DTT) at a concentration of 1 × 10^8^ nuclei/ml and washed and centrifuged two more times in buffer D. An equivalent of 1 × 10^7^ nuclei/ml was resuspended in buffer MN (60 mM KCl; 15 mM NaCl; 15 mM Tris, pH 7.4; 0.5 mM dithiothreitol (DTT); 0.25 M sucrose and 1 mM CaCl_2_). Aliquots of 100 or 200 ul were digested with 200 U MNase for 10 min at 37°C, adding 400 ug RNase A for the last 3 min. Digestion was stopped by adding EDTA and sodium dodecyl sulphate to a final concentration of 12.5 mM and 0.5%, respectively. DNA was then isolated using Qiagen DNeasy kit and checked for complete digestion of the genomic DNA into mononucleosomes.

#### Real Time quantitative polymerase chain reaction (qPCR) analysis

Primers were designed to amplify 100 ± 8 bp amplicon sizes, overlapping by 30 ± 10 bp. Primer pair efficiencies were determined to be within 0.5 cycles of each other. A total of 633 bp of the promoter plus 5′ region and 509 bp of the transcribed 3′ end of the firefly luciferase gene contained in the roX2, and Nesprin plasmids were included in the analysis. Real-time qPCR (QRT-PCR) was performed on equal amounts of undigested and MNase-digested DNA. As described in Petesch and Lis (34), the percentage protection was calculated by comparing *C*_t _values of undigested versus MNase-treated samples for each primer pair. This was done in triplicate.

## RESULTS

### H4K16ac disorganizes chromatin fibers

**Figure 1.**
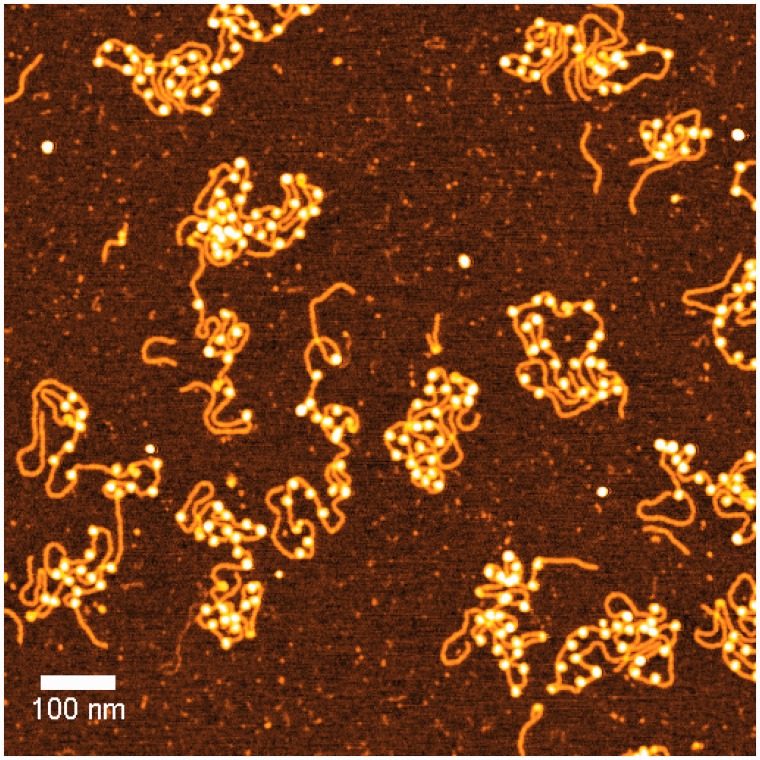
A representative atomic force micrograph of chromatin reconstituted with recombinant *D. melanogaster* histones and a 5049-bp DNA template with 24 repeats of a 177-bp sequence containing the 601 nucleosome positioning sequence. Most molecules have a regular array of histone octamers.

The tail of histone 4 was found to interact with the H2A/H2B surface of the neighboring nucleosome in X-ray crystal structures (8). Acetylation of lysine 16 of histone H4 (H4K16ac) weakens interactions between neighboring nucleosomes such that precipitation is discouraged (22). In addition, H4K16ac weakens the cation-induced condensation of reconstituted nucleosomes (23). These data suggested that H4K16ac may profoundly modify the structure of the 30-nm chromatin fiber. To investigate this possibility, chromatin was reconstituted on DNA templates containing 24 repeats of a 177-bp segment that includes the 601 nucleosome positioning sequence. The B-form length of this naked DNA was ∼1700 nm, but when reconstituted with histone octamers as chromatin, the tethers ranged from 400 to 1300 nm in length, presumably depending on the degree of octamer loading. Indeed, using similar reconstitution conditions, experiments with a segment of DNA containing an array of twelve 601 repeats, each 177 bp in length, revealed octamer loading that ranged from 5 to 12 with a median of 10 (data not shown). However, 60% of those molecules had ≥10 octamers; therefore, most single 24-repeat molecules were likely to have a sufficient octamer density to exhibit the interactions expected in a chromatin fiber (see Figure 1).

In solution, polymers like DNA and chromatin are buffeted by thermal energy and bend into randomly coiled configurations. The coiled state with many possible configurations has much higher entropy (lower free energy) than a fully extended configuration. Therefore, polymers tend to prefer randomly coiled states, and behave as retractile ‘entropic’ springs that resist stretching. Softer polymers with shorter persistence lengths are more disordered, and are stronger ‘entropic’ springs than relatively stiffer polymers with longer persistence lengths (Supplementary Figure S1). These random coils can be modeled as smoothly bent, ‘worm-like’, chains, for which the length scale of bending is indicated by the persistence length, the largest separation between two points along the polymer for which the intervening segments are, on average, straight or only slightly bent. ‘Stiff’ polymers have long lengths over which the direction of the chain persists, whereas ‘soft’ polymers bend after only a short distance along the contour. To determine whether H4K16 acetylation weakens nucleosome interactions and increases the random coiling of a chromatin fiber, single DNA and reconstituted chromatin molecules were gently stretched, and the tension versus extension data were fit using a worm-like chain expression to determine persistence length (25). An example of the data is provided in Supplementary Figure S2.

**Figure 2.**
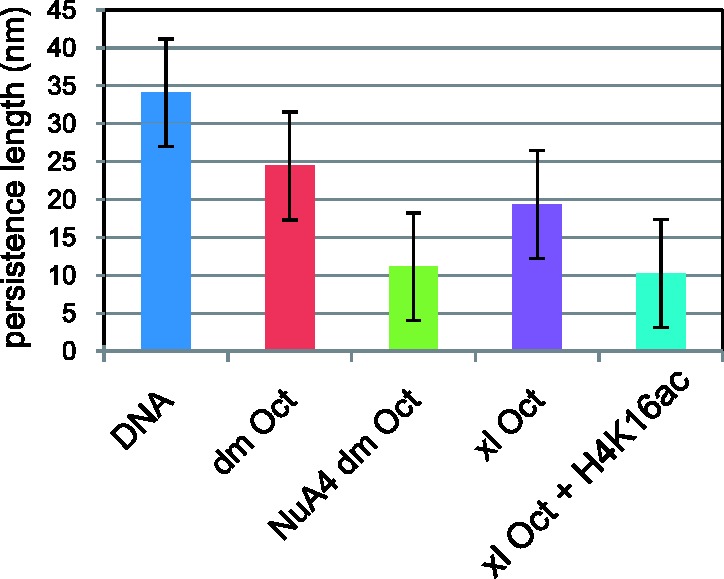
Persistence length values were determined from fits to tension versus extension data recorded for DNA and reconstituted chromatin. Unacetylated chromatin, reconstituted with *Drosophila *(*dm* Oct) or with *Xenopus* (*xl* Oct) octamers, was a stronger entropic spring with a shorter persistence length and a higher force constant than the bare DNA filament (DNA), but acetylation by either NuA4 (NuA4 *dm* Oct) or reconstitution with acetylated H4K16 (*xl* Oct + H4K16ac) produced even stronger entropic springs with even shorter persistence lengths. The extension was recorded as the tension was increased in steps from tens of femtonewtons to near ten piconewtons. Error bars represent standard deviations.

Force versus extension curves for both DNA and chromatin were well fit up to 10 pN of tension beyond which unfolding occurred. As shown in simulations, such low tension straightens entropic bends and begins to weaken internuclesomal interactions (35,36). In this low-tension regime, slight weakening of the fiber makes it more susceptible to bends induced by thermal energy. Increased bending is associated with shorter persistence length and more randomly coiled fibers that act as stronger entropic springs. Although DNA devoid of nucleosomes exhibited a persistence length of 34 ± 9 nm, chromatin reconstituted with either recombinant histones of *D. melanogaster* or *X. laevis* had significantly shorter persistence lengths of 22 ± 9 or 19 ± 8 nm (Figure 2, Supplementary Table S1). Reconstituted chromatin that was acetylated nonspecifically on H2A and H4 by the Piccolo subcomplex of the NuA4 (nucleosome acetyltransferase of H4) complex (37) or reconstituted with octamers containing H4 synthetically acetylated on lysine 16 (23) had even shorter persistence lengths of 11 ± 6 and 10 ± 7 nm indicative of weaker fibers. As stated earlier, relatively shorter persistence length correlates with higher entropy and greater disorder. Such a correlation between greater disorder and lower persistence length was shown in simulations in which chromatin fibers were modeled as a chain of nucleosomes with constant entry/exit angles of the linker DNA, whereas the twist angle between successive nucleosomes was allowed to vary randomly around a central value of 110°. Expanding the range of variation from 10 to 30° created more irregular fibers and caused the persistence length to drop from 240 to 24 nm (38). Similarly, our molecular force spectroscopy data suggest that acetylation of H4K16 alone can significantly weaken nucleosome packing in a chromatin fiber lowering the persistence length and presumably facilitating access to and enhancing the transcription of certain genes.

### Histone acetylation does not contribute to the topological changes exhibited by compensated chromatin

**Figure 3.**
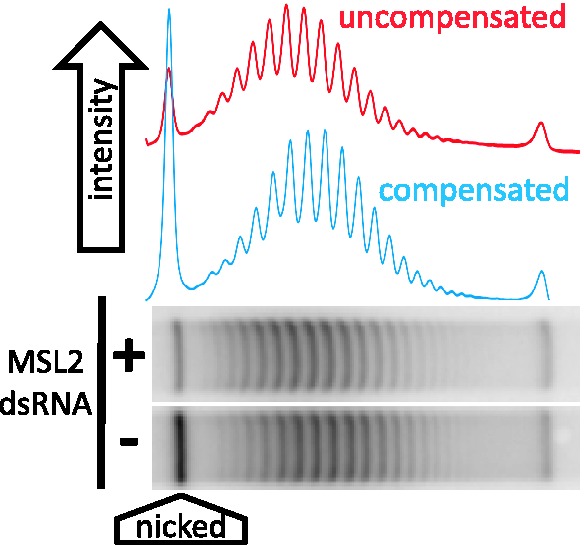
A representative gel of topoisomers isolated from S2 cells treated with RNAi to green fluorescent protein (GFP) [mock, (−) in the figure] or to a component of the MSL complex. A high concentration of chloroquine was added to plasmids isolated from the cells to resolve topoisomer distributions by gel electrophoresis. Plasmids isolated from ‘compensated’ cells (GFP) migrate further in this gel indicating a lower level of negative supercoiling.

We have used an experimental system consisting of a plasmid with a DNA fragment from the *roX2* gene that nucleates the MSL complex and results in a 2-fold enhancement of transcription of a firefly luciferase reporter gene (33). Recently, we have compared the topoisomer distributions of plasmids extracted from S2 cells in which the MSL complex forms and, therefore, the reporter gene on the plasmid is dosage compensated, with plasmids extracted from cells in which the complex is knocked down by RNA interference (noncompensated or ‘reference’ plasmids). Our results showed that the compensated plasmid is less negatively supercoiled, and that this difference is not because of the enhanced transcription mediated by dosage compensation (Cugusi *et al.*, submitted for publication). An example of this data is presented in Figure 3. To determine whether the acetylation of histone H4 contributes to the topological changes exhibited by the plasmid in cells where the MSL complex is present, we repeated the experiments in the presence of the histone deacetylase inhibitor trichostatin A (TSA). Treatment with TSA led to an ∼4-fold increase in the level of H4 acetylation in S2 cells (Supplementary Figure S3A) without altering the difference in the linking number caused by the presence of the complex. In the absence of TSA, the difference in the linking number between the topoisomer distribution of a plasmid subjected to dosage compensation and a control plasmid is 1.48 ± 0.4 (*P=*0.002), whereas in the presence of TSA, the difference is 1.57 ± 0.48 (*P=*0.002); these two values are not significantly different (*P=*0.39). TSA treatment increased the level of H4K16 acetylation to a small extent (Supplementary Figure S3B). Therefore, to determine whether this specific modification is responsible for the change in topology of the dosage compensated plasmid, we depleted MOF in S2 cells. The results showing the effect that H4K16ac had on the level of supercoiling of the plasmid are described later.

### Changes in negative supercoiling are not reflected in nucleosome loss

**Figure 4.**
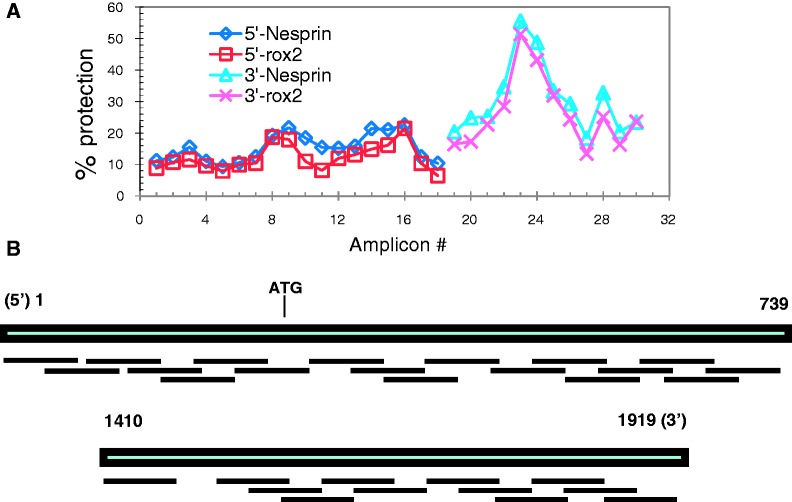
Nucleosome mapping on the compensated and reference firefly luciferase genes. (**A**) MNase protection profile determined from QRT-PCR cycle numbers. (**B**) Diagram depicting the PCR amplicons used to map the positions of 5′ and 3′ nucleosomes in the firefly gene. Numbering starts at the 5′ end of the gene.

In chromatin, each nucleosome protects one negative supercoil in the DNA with which it is associated from the action of endogenous topoisomerases. A change in the total negative superhelicity of a plasmid following extraction may result from the loss of nucleosomes or from an alteration in the DNA—nucleosome association allowing an opportunistic access to the DNA by a topoisomerase. Although the difference in linking number between a compensated and a noncompensated plasmid is small, we wished to determine whether it represented the loss of a particular nucleosome. We used an MNase and real-time qPCR-based method to map nucleosomes along two segments of the firefly luciferase gene in a compensated and a reference plasmid (34,39). There does not appear to be any nucleosome loss from the promoter and 5′ or the 3′ ends of the compensated gene (Figure 4). The results seem to indicate a general (albeit not significant) trend of increased sensitivity to MNase digestion of the compensated plasmid.

### Partial MSL complexes contribute incrementally to the topological characteristics of compensated chromatin

**Figure 5.**
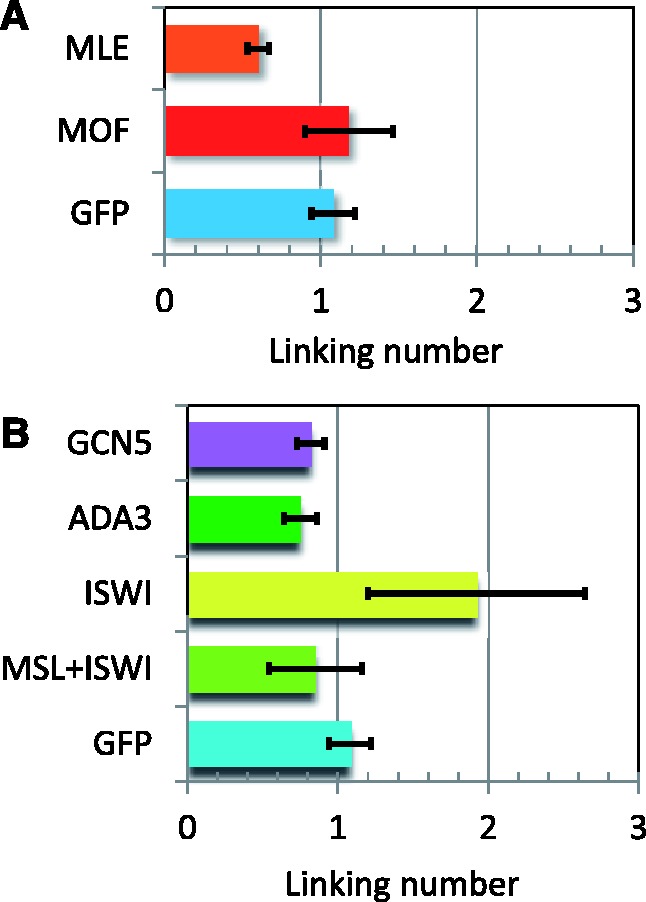
The differences in the median linking number of topoisomers isolated from S2 cells treated with RNAi to knockdown the indicated proteins were determined with respect to the linking number of plasmids isolated from cells in which MSL2, and therefore the MSL complex, was absent. (**A**) MOF knockdown, which leaves a complex of MSL1 + MSL2 + MLE, did not significantly change the topology compared with plasmid from compensated cells (GFP). MLE knockdown, which leaves a complex of MSL1 + MSL2, resulted in a partial or intermediate reduction in negative supercoiling of the plasmid. (B) ISWI knockdown produced more relaxed noncompensated and compensated topoisomers that migrated one linking number ahead of the similar plasmids from untreated cells. However, ADA3 and GCN5 knockdown in S2 cells did not significantly change the topology nor the migration of the isolated topoisomers.

Using RNA interference, we selectively eliminated components of the MSL complex (Supplementary Figure S4). Assembly of the entire complex is prevented by the absence of MSL2 (40). In the absence of MOF, only MSL1 + MSL2 + MLE associate with the chromatin entry sites of the complex (41); in the absence of MLE, only MSL1 + MSL2 are at these sites (42). In the current set of experiments, the linking number difference between a compensated plasmid extracted from S2 cells that form a fully functional complex and a plasmid extracted from cells where the complex is absent was 1.08 ± 0.14 (*P=*0.0003; see Figure 5A). The mobility of the topoisomer distribution of a plasmid extracted from cells where a partial complex composed of MSL1 + MSL2 + MLE was similar to that of the fully compensated plasmid; the difference in the linking number between these two plasmids was 0.11 ± 0.29 (*P=*0.26). The linking number of a plasmid extracted from cells in which MSL1 + MSL2 are the only subunits that associate with the chromatin entry site differed from that of the noncompensated plasmid by 0.6 ± 0.07 (*P=*0.002). Therefore, under our experimental conditions, this plasmid exhibited a partial relaxation of negative supercoils. These data suggest that MOF, and therefore H4K16 acetylation, and MSL3 do not contribute to the topological changes exhibited by a plasmid exposed to a complete MSL complex. A partial complex made up of MSL1, MSL2 and MLE was sufficient to produce these changes. Finally, just the presence of MSL1 and MSL2, the two MSL subunits that bind DNA, was sufficient to partially alter the topology of the plasmid.

### Interaction of the MSL complex with general chromatin regulators

**Figure 6.**
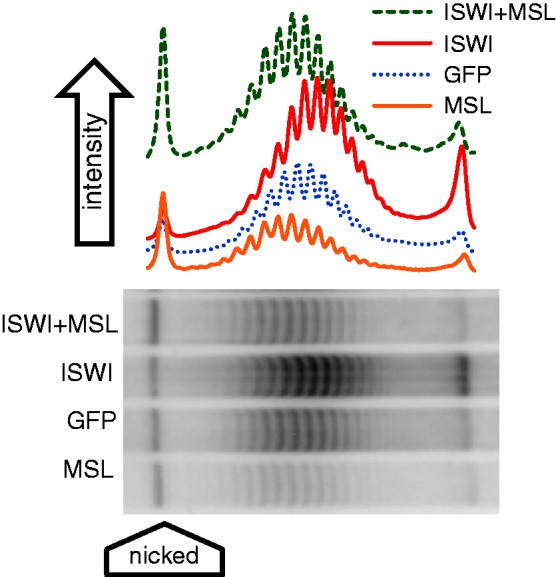
Representative distributions of topoisomers isolated from S2 cells treated with RNAi to MSL, GFP (mock), ISWI or ISWI + MSL. A high concentration of chloroquine was added to plasmids isolated from the cells to resolve topoisomer distributions by gel electrophoresis.

We used the plasmid system to determine whether the gross morphological changes in chromosome morphology observed as a result of diminished ISWI function were reflected in the topological characteristics of the DNA. An example of this data is presented in Figure 6. Cells transfected with *roX2* sequence-bearing plasmids, treated with double-stranded MSL2 RNA to prevent the formation of the MSL complex or with GFP RNA, were subjected to RNA interference against ISWI leading to a reduction of 75% of the transcript as measured by QRT-PCR. Knockdown of ISWI in cells where the MSL complex is absent resulted in a difference in linking number of 0.85 ± 0.31 (*P=*5 × 10^−^^6^). This reflects the normal effect of ISWI on the topology of the reference (noncompensated) plasmid. ISWI knockdown in cells in which the MSL complex is present altered the topology of the compensated plasmid by a difference in linking number of 1.92 ± 0.72 (*P=*0.006), in comparison with the reference plasmid. This change in the linking number represents the difference in topology normally present between compensated and noncompensated plasmids (∼1 linking number) plus the effect of ISWI knockdown on the topology of the nondosage-compensated reference plasmid (0.85 ± 0.31 in the present experiment) and indicates that ISWI does not contribute to the reduction of negative supercoils of compensated transcriptional units. In support of this conclusion is the fact that the differences in linking number caused by ISWI knockdown in a compensated (1.16 ± 0.59; *P=*0.016) and noncompensated plasmid (0.85 ± 0.31; *P=*5 × 10^−^^6^ as indicated earlier) are equivalent (*P=*0.27). These comparisons are summarized in Figure 5B.

**Figure 7.**
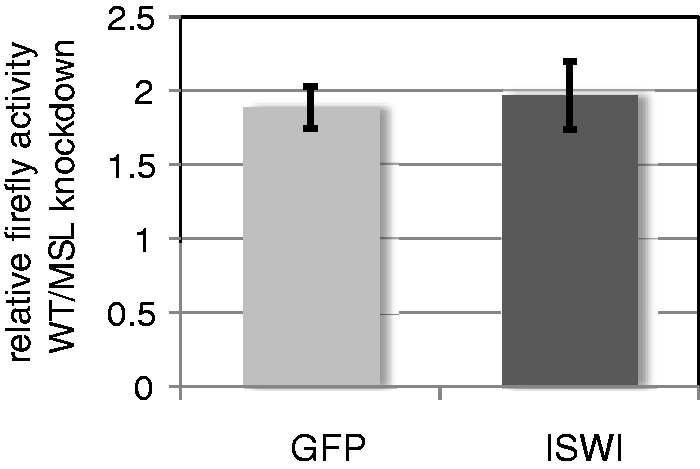
Dosage compensation was measured as the ratio of relative luciferase activity (firefly vsersus *Renilla*) between S2 cells transfected with plasmid bearing a roX site next to the firefly gene and S2 cells transfected with a plasmid lacking a *roX* site. In S2 cells treated with RNAi to GFP (mock) or ISWI, ISWI knockdown did not affect dosage compensation.

The absence of an effect of ISWI knockdown on the change in template topology specifically associated with dosage-compensated genes led us to ask whether dosage compensation of the plasmids, as determined by the relative activity of the firefly luciferase reporter gene, remains unchanged. Firefly luciferase activity relative to *Renilla* luciferase activity was measured in cells in which the MSL complex forms and in cells in which it does not, in the presence or absence of ISWI RNA interference. The absence of ISWI leads to a substantial increase in the transcription of the firefly luciferase gene in both types of cells, whereas the *Renilla* luciferase gene exhibits only a minor enhancement in transcription (data not shown). Nevertheless, the results of these experiments show that the absence of ISWI has no effect on the dosage compensation of the firefly plasmid (Figure 7).

Mutations in *Gcn5 *(general control nonderepressible 5)*, Ada3* or *Ada2a* (adaptor protein 3 or 2a) cause a defect, similar to that induced by mutations in the *Iswi* gene, in the banding organization of polytene chromosomes in both males and females, with more severe effects on the X chromosome in males. GCN5 and ADA3 are components of the SAGA (Spt-Ada-Gcn5-acetyltransferase) and ATAC (Ada two A containing) complexes that acetylate histones; ADA2a is found only in the ATAC complex (43,44). The complete identification of the ATAC complex subunits revealed the presence of a second histone acetyl transferase, ATAC2, that specifically contributes to the acetylation of H4K16; furthermore, the ATAC complex stimulates nucleosome sliding by the ISWI (imitation switch), SWI/SNF (switch/sugar nonfermenter) and RSC (remodel the structure of chromatin) complexes, although it has no remodeling activity of its own (45). To determine whether ATAC or SAGA contribute to the topology of dosage-compensated transcriptional units, we used RNA interference to deplete S2 cells of GCN5 or ADA3 (Supplementary Figure S4). Knockdown of GCN5 and ADA3 increased the linking number of the compensated plasmid by 0.17 ± 0.08 and 0.24 ± 0.11, respectively (Figure 5B). Neither of these differences is significant, suggesting that these two complexes are not involved in the topological changes exhibited by compensated chromatin.

## DISCUSSION

We have tested the role of H4K16ac on internucleosomal interactions by measuring the force necessary to gently stretch a single chromatin fiber reconstituted with unmodified histones or with histone H4 acetylated at lysine 16. As a positive control, we showed that a fiber that is acetylated by the Piccolo subcomplex at several lysines on histone H2A and H4, including H4K16, is weaker than an unmodified fiber. We repeated these experiments by reconstituting chromatin fibers with unmodified histones H2A, H2B and H3 and with chemically synthesized H4 acetylated at lysine 16. Our results clearly indicate that the H4K16ac modification suffices to weaken the chromatin fiber. Shogren-Knaak *et al.* found that H4K16 acetylation interfered with Mg^+2^-induced compaction and aggregation of nucleosomal arrays (22). Our stretching experiments show that magnesium is not essential for H4K16-dependent internucleosomal interactions. A possible correlation between this structural effect of acetylation and enhanced transcription may be provided by the observation that the presence of H4K16ac increases accessibility for DNA-binding extrinsic proteins (46).

The structural role of H4K16ac does not rule out additional potential mechanisms based, for instance, on the recognition of this modification by chromatin-associated proteins harboring one or more bromodomains. In human cells, H4K16ac has been shown to recruit BRD4 (bromodomain-containing protein-4) to release RNAPII from pausing (47,48). BPTF (bromodomain PHD finger transcription factor), a subunit of the NURF ATP-dependent chromatin remodeling complex, is recruited to chromatin by the combination of methylated H3K4me and H4K16ac (49). Emphasizing the likelihood that other factors may contribute to the mechanism of dosage compensation is the recent screen based on RNA interference that identified a large number of potential MSL complex interactors (50).

Additional parameters, independent of the role played by H4K16ac, are clearly involved in the molecular mechanism responsible for dosage compensation. Notably, we have shown that a transcriptional unit exhibits fewer negative supercoils when it is dosage compensated, that the loss of negative supercoils in compensated chromatin is correlated to the recruitment of topoisomerase II and have suggested that this is mediated by the MLE subunit of the MSL complex (Cugusi *et al.*, submitted for publication).

The full reduction in negative supercoils exhibited by the *roX* sequence-bearing plasmid when MSL1 + MSL2 + MLE are present reinforces the conclusion that the acetylation of H4K16 is not necessary for this topological modification. Surprisingly, the presence of only MSL1 + MSL2 yielded a partial reduction in the negative supercoiling of the roX-bearing plasmid. This observation can be explained by considering that the plasmid introduced in S2 cells is fully relaxed. As the plasmid DNA wraps itself around histone octamers, it undergoes negative supercoiling. Binding of the MSL1–MSL2 heterotetramer to the *roX *sequence may interfere to some extent with the total number of supercoils that could be generated. Conceivably, the presence of MSL1 + MSL2 may be sufficient to alter the topoisomerase activity that is normally involved in the chromatinization of the plasmid.

Attempts to determine the possible function of the ATPase ISWI in dosage compensation illustrate the difficulty in distinguishing the role of various factors on the causal mechanism of dosage compensation (the chromatin modifications that cause the 2-fold enhancement of transcription) from the resulting effect of this mechanism (the actual occurrence of enhanced transcription). The former are chromatin modifications that occur on particular X-linked genes in males. The latter involves factors that are responsible for transcription of these and all other RNAPII-dependent genes in both males and females. ISWI is the ATPase common to the CHRAC, ACF, NURF and RSF chromatin remodeling complexes. Although a number of *in vitro* studies have indicated that some ISWI complexes establish ordered nucleosomes, whereas others disrupt nucleosomal arrangements in chromatin fibers, it is clear that *in vivo*, their functions depend on histone modifications and interactions with other chromatin-modifying and remodeling complexes. For example, the association of ISWI with a nucleosome is impaired by the presence of acetylated lysines 12 and 16 on the H4 tail (51,52). Similar results were obtained specifically for the ISWI-containing NURF complex (53). Furthermore, the nucleosome sliding promoted by remodeling complexes, including ISWI, is enhanced by the function of the histone acetyltransferase ATAC complex (45).

In the absence of ISWI, both control and compensated plasmids were more relaxed and, therefore, ran faster in our experimental conditions. In the case of the compensated plasmid, this effect was amplified by the fact that it is already more relaxed (Cugusi *et al.*, submitted for publication). These observations lead us to conclude that the presence of ISWI normally affects the topological organization of chromatin in general without any particular effect on dosage-compensated regions.

Expression of a dominant negative form of ISWI leads to a global loss of polytene chromosome structure that is not limited to the X chromosome in males as has been described previously using heteroallelic combinations of partial loss-of-function mutant alleles. The loss of ISWI results in a change in the transcription level of target genes, with ∼75% of the genes exhibiting enhanced transcription; these genes are randomly distributed between the X chromosome and the autosomes (54). A more recent study established the genome-wide binding of ISWI and the changes in nucleosome positioning following ISWI knockdown (55). Although there is a low level of global binding, ISWI is enriched in the region immediately following the nucleosome-free transcription start site (TSS) where it appears to position nucleosomes. The presence of ISWI is greater on the X chromosome in both males and females, but its loss causes changes in nucleosome spacing after the TSS that are more pronounced in males than in females. On the male X chromosome, genes that are strongly bound by the MSL complex present a positioned nucleosome in the pre-TSS region; loss of ISWI results in the delocalization of this nucleosome and allows it to occupy the TSS. Although genes that are not bound by the MSL complex in males, and X-linked genes in females show poorly positioned nucleosomes throughout the TSS, the absence of ISWI results once again in changes in nucleosome positioning. All of these observations point to the possibility that ISWI may play a regulatory role on the level of expression of dosage-compensated genes. The increase in the level of transcription of many genes in the absence of ISWI (54) suggests that its presence may serve as a monitor to maintain gene activity at the appropriate level, perhaps by modulating the association of histone H1 (56). This may be particularly important in the case of genes on the X chromosome in males that are subjected to the mechanism of dosage compensation designed to increase their activity, but only to an approximate 2-fold level. The presence of an excess of ISWI on both male and female X chromosomes may be because of the selection of more binding sites on this chromosome because of its function in dosage compensation. In females, the action of ISWI may be regulated on the X and throughout the active genome by some limited cofactor. Particular features of compensated genes, such as the difference in superhelicity of their DNA, may facilitate the activity of ISWI in males. In this respect, the conflicting functions of H4K16ac and ISWI demonstrated by several *in vitro* experiments would be avoided on compensated genes: H4K16ac occurs increasingly toward the 3′ end and is relatively sparse around the TSS where ISWI appears to operate (15,16).

Although depletion of ISWI by RNA interference or by mutation will affect the function of the four distinct chromatin remodeling complexes mentioned earlier, some published observations imply a specific role for NURF (57). In a mutant *nurf* background, loss-of-function mutations in either *roX1* or *roX2* lead to a more normal appearance of the polytenic X chromosome in the general region of the mutation; conversely, a wild-type *roX* transgene relocated to an ectopic autosomal location nucleates a region of disorganization at its site of insertion. Furthermore, the NURF complex prevents the transcription of roX RNAs in females and the overtranscription of *roX2* in males.

Mutations in subunits of the SAGA and ATAC complexes, which have been shown to result in a decondensation of polytene chromosomes in males and females and especially of the X chromosome in males, have no effect on the superhelicity of a compensated plasmid. This apparent lack of effect on dosage compensation is supported by the observation that genome-wide profiling did not show a disproportionate set of X-linked genes among the genes affected in *Gcn5* or *Ada2a* mutants (44).

Chromatin remodeling and -modifying enzymes usually do not function individually. They are included in complexes consisting of multiple subunits that often perform different functions. The reason for this organization may be that it allows for the rapid coupling of reactions (58). The MSL complex mediates two chromatin alterations through the function of different subunits: the acetylation of histone H4 at lysine 16, which disorders the chromatin fiber, and a reduction in the level of negative supercoiling that may facilitate the elongation phase of transcription.

## SUPPLEMENTARY DATA

Supplementary Data are available at NAR Online: Supplementary Table 1, Supplementary Figures 1–4, Supplementary Methods and Supplementary References [59–62].

## FUNDING

National Institutes of Health [GM15691 to J.C.L.]; Human Frontiers Science Program [RGP0051/2009 to D.D.]. Funding for open access charge: NIH [GM15691 to J.C.L.].

*Conflict of interest statement*. None declared.

## Supplementary Material

Supplementary Data
