# Supplementary material for: Distinct contributions of MSL complex subunits to the transcriptional enhancement responsible for dosage compensation in Drosophila
Source: Nucleic Acids Res. 2012 Oct 9;40(22):11281–91. doi: 10.1093/nar/gks890 (PMC3526317; doi:10.1093/nar/gks890)
Supplement: Supplementary Data [file supp_40_22_11281__index.html]

Distinct contributions of MSL complex subunits to the transcriptional enhancement responsible for dosage compensation in Drosophila — Distinct contributions of MSL complex subunits to the transcriptional enhancement responsible for dosage compensation in Drosophila — Supplementary Data 

# Distinct contributions of MSL complex subunits to the transcriptional enhancement responsible for dosage compensation in *Drosophila*

## Supplementary Data

files

**Files in this Data Supplement:**

- Supplementary Data - pdf file
