# Supplementary material for: Distinct contributions of MSL complex subunits to the transcriptional enhancement responsible for dosage compensation in Drosophila
Source: Nucleic Acids Res. 2012 Oct 9;40(22):11281–91. doi: 10.1093/nar/gks890 (PMC3526317; doi:10.1093/nar/gks890)
Supplement: Supplementary Data [file supp_gks890_nar-01596-x-2012-File002.pdf]

SUPPLEMENTARY METHODS

Primers used to synthesize double-stranded RNA

MLE forward 5' CCATAGAGAATGCCAAAGAGC 3'
reverse 5' ATCAATAGAGGTGGTTGCCAG 3'
GFP forward 5' ACGTAAACGGCCACAAGTTC 3'
reverse 5' TGCTCAGGTAGTGGTTGTCG 3'
MSL2 forward 5’ TTCACAGTCACCTACACAAACC 3’
reverse 5’ CTCGCCTTTCTCATTCTGAAC 3’
MOF forward 5’ AGCCGCAGAAGCAGAAAG 3’
reverse 5’ CCATGGTCAAGGCATCATC 3’
ISWI forward 5’ CCAAGTGTTCAGCTCTAAGGAG 3’
reverse 5’ TTGGTCCAGGCAGTGAAAC 3’
Ada3 forward 5’ CATGAGTGCGAACCTGAAGA 3’
reverse 5’ GAACTTGTTGGGGGTATCGT 3’
Gcn5 forward 5’- AACAGCAGCAGCAGGCAGCGAATGG 3’
reverse 5’ CACTGGCGCAGCAAACGGAAGAGGT 3’

Preparation of flow chambers

No. 1 50 X 22 mm coverglasses were cleaned with soap, rinsed copiously with tap water, and then submerged for at least 30 min with gentle agitation in ethanol in which they were stored until use. Before use the ethanol was drained and then dried from each coverslip with lab tissue and an aerosol duster. A serpentine channel was cut in a piece of parafilm which was placed between the crossed coverglasses and heated enough to seal the parafilm to the glass with gentle pressure. The channel was rinsed with 100 μl of 0.2 mm filtered PBS and then 50 μl of 20 mg/ml anti-digoxigenin diluted in PBS (Roche) was introduced into the chamber and incubated at 100% relative humidity overnight at 4 degrees C. After 30 sec of vortexing, 1 μl of MyOne beads (Invitrogen, Carlsbad, CA) were washed three times by resuspension in 150 μl of 1 M NaCl in TE, 5 min of magnetic precipitation, and removal of the solution. The washed beads were finally resuspended in 150 μl of 0.2 μm filtered PBS and introduced into the chamber which had been flushed with two volumes of filtered PBS and incubated at least 10 minutes. This first aliquot of beads adhered directly to the surface and served as reference beads. An additional 2 μl of MyOne

beads were washed three times as above. These washed beads were finally resuspended in 100 μl of 100 mM NaCl in TE. 2 μl (about 50 ng total DNA) of the ligation reaction was added to 100 μl of 100 mM NaCl in TE and mixed with the resuspended microspheres in a 1.5 ml microcentrifuge tube for a ten minute incubation with gentle flicking every minute to prevent bead sedimentation. The microchamber was rinsed with three volumes of 100 mM NaCl, 0.2 mg/ml casein w/ 0.5 % Tween in 20 mM Tris-HCl pH 7.4 and incubated for at least 5 min to passivate any remaining exposed glass in the chamber. The bead-DNA solution was spiked with 4 ml of 0.1 mM d-biotin (Sigma) in TE, mixed, and incubate for 5 min. 50 μl of the bead-DNA solution was introduced into the microchamber. After 5 min. the microchamber was gently rinsed with three volumes of 100 mM NaCl with 0.2 mg/ml casein w/ 0.5 % Tween in 20 mM Tris-HCl pH 7.4 to remove unbound untethered beads.

Force extension measurements

Stretching reconstituted chromatin has shown that histone octamers release wrapped DNA in two stages as a function of tension (59). The external turn of DNA easily dislodged at tensions below <15 pN while unwrapping the internal turn of DNA centered on the dyad axis required more tension. H4K16 acetylation is not expected to affect the wrapping of DNA on the histone octamers, but the association of neighboring octamers. Therefore stretching experiments were carried out using tensions below 10 pN. At such tensions, Kruithof and colleagues have shown a Hookeian spring-like behavior for arrays of twenty-five 601 repeats, each 197 bp in length (60). Instead the low force vs. extension curves for chromatin arrays of twenty four repeats, each 177 bp in length, were typically smooth curves (Fig. S2) much like those reported for chromatin fibers purified from chicken erythrocytes (61). Whether or not chromatin reacts as a Hookeian or entropic spring may be a function of the rotational freedom of the fiber. DNA nicks, linkages relying on single biotin- or digoxigenin-labels, and optically trapped beads may swivel to relax torque. Although chromatin fibers have been shown to absorb torque applied using magnetic tweezers (62), and DNA the fibers in the stretching experiments described here was presumably intact since it was prepared using ligase, these fibers were not deliberately twisted.

SUPPLEMENTARY RESULTS

t-test (%)	DNA	*dm* Oct	NuA4 *dm* Oct	*xl* Oct
dm Oct	1.9			
NuA4 *dm* Oct	0.0	2.0		
xl Oct	0.6	48.8	1.4	
xl Oct + H4K16ac	0.0	0.1	72.7	0.8

Table S1. T-tests were used to estimate the significance of differences between persistence lengths determined for fibers during stretching. The probabilities (%) that two sets of measurements compared derive from the same populations are listed. Persistence lengths were not significantly different when reconstituted chromatin was prepared with recombinant histones corresponding to either *Drosophila melanogaster* (*dm* Oct) or *Xenopus laevis* (*xl* Oct). Chromatin prepared with either synthetic H4K16ac (*xl* Oct + H4K16ac) or acetylated using NuA4 *in vitro* (NuA4 *dm* Oct) was also indistinguishable. However, both types of acetylated chromatin were significantly different from unacetylated chromatin and DNA.

SUPPLEMENTARY FIGURES

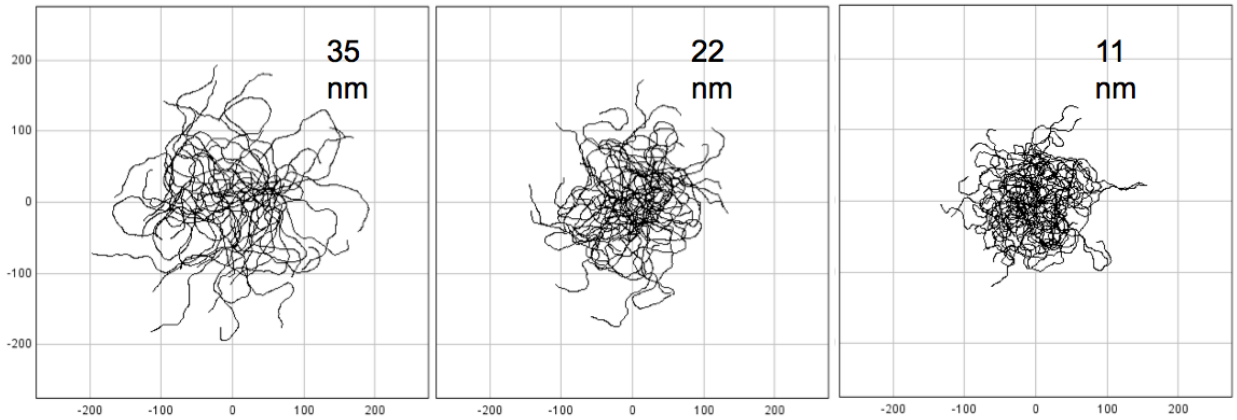

Supplementary Figure S1. Stronger "entropic" springs have lower persistence lengths. 20 examples of worm-like chains (WLCs) with persistence lengths of 35, 22, or 11 nm (left, middle and right respectively) were simulated and plotted using a common origin. The average extension of these random coils in their lowest energy conformation is inversely related to the persistence length. To stretch them all to the same average extension would require added tension for the WLCs with 22 nm persistence length and even more for the WLCs with 11 nm persistence length.

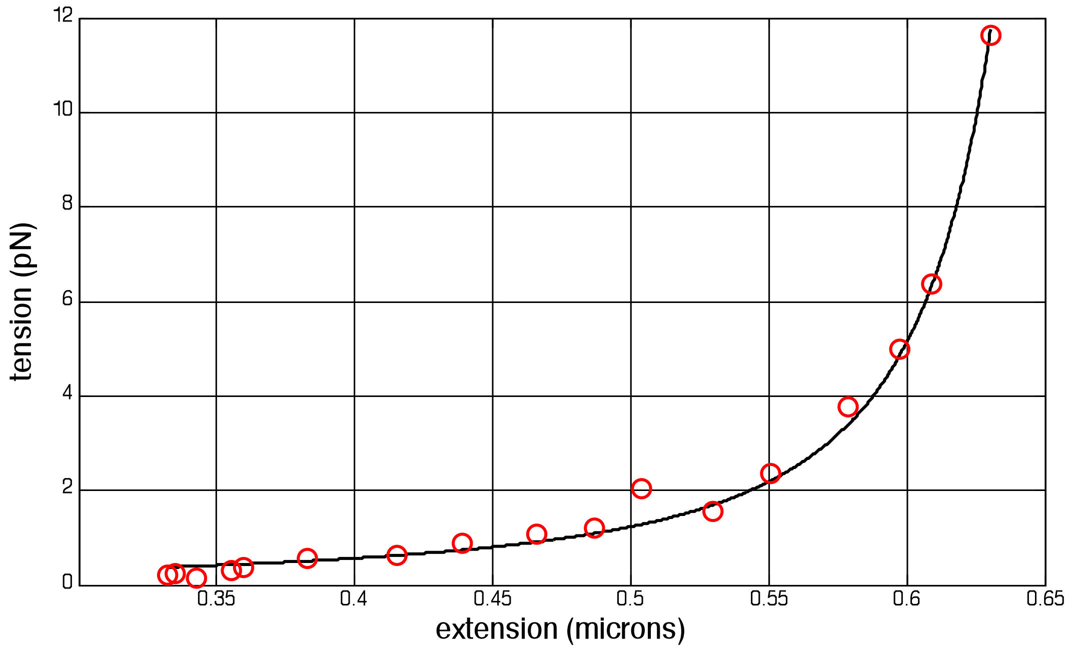

Supplementary Figure S2. A representative tension versus extension curve for a chromatin fiber acetylated on H4K16. The red circles are measured data and the black curve is a worm-like chain fit.

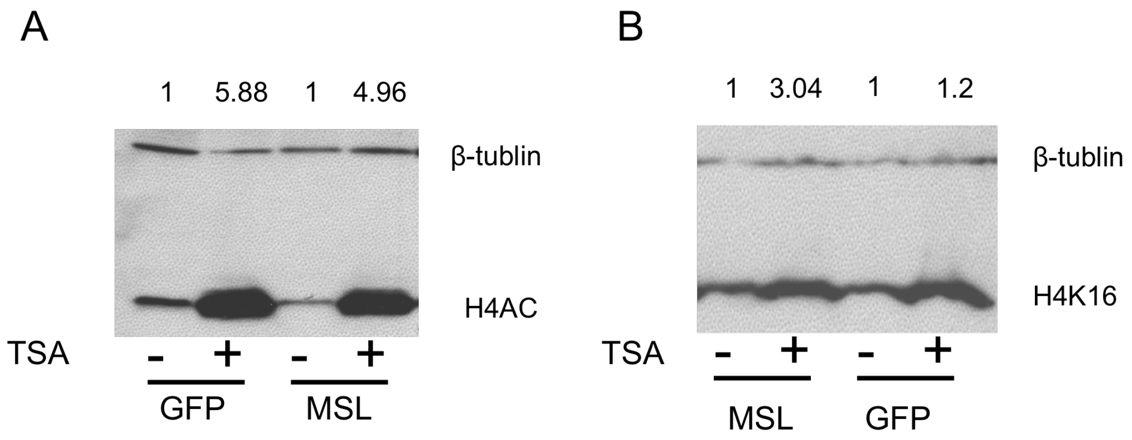

Supplementary Figure S3. Trichostatin A was administered to S2 cells as described in the material and methods and the extent of acetylation of (A) histone 4 (H4ac) or (B) lysine 16 of histone 4 (H4K16ac) was determined by immunoblotting. The numbers represent quantitative scanning of the gels.

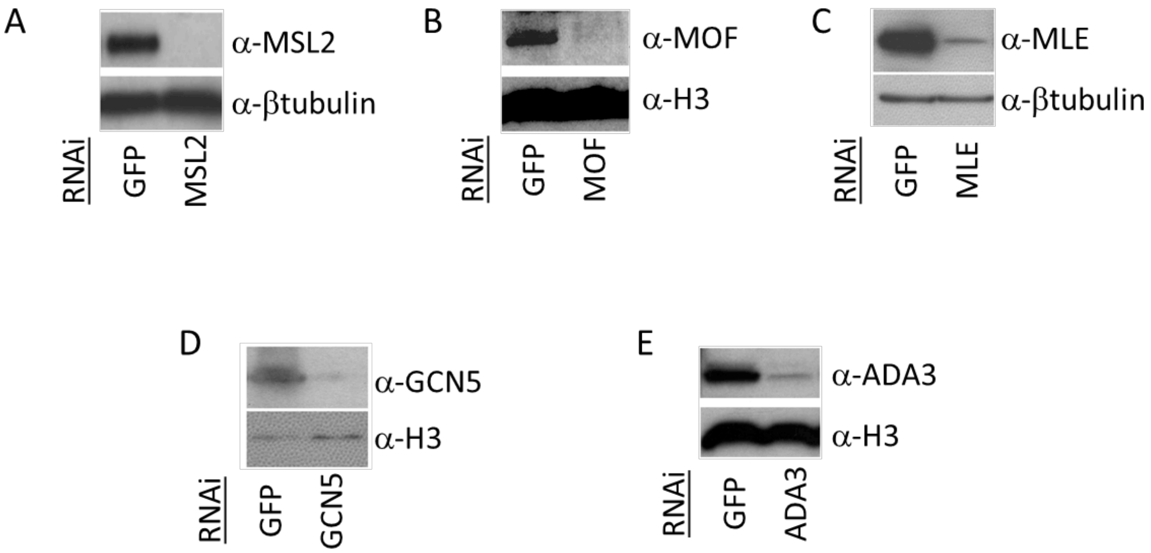

Supplementary Figure S4. Immunoblotting was used to verify decreased levels of proteins in S2 cells treated with dsRNA. S2 cells were treated with dsRNA to knockdown the levels of components of the MSL complex. Significantly lower levels of (A) MSL, (B) MOF, (C) MLE, (D) GCN5, and (E) ADA3 were evident while the levels of *β*-tubulin or histone 3 (H3) used as controls did not change.

SUPPEMENTARY REFERENCES

59. Brower-Toland, B.D., Smith, C.L., Yeh, R.C., Lis, J.T., Peterson, C.L. and Wang, M.D. (2002) Mechanical disruption of individual nucleosomes reveals a reversible multistage release of DNA. *Proceedings of the National Academy of Sciences of the United States of America*, **99**, 1960-1965.
60. Kruithof, M., Chien, F.T., Routh, A., Logie, C., Rhodes, D. and van Noort, J. (2009) Single-molecule force spectroscopy reveals a highly compliant helical folding for the 30-nm chromatin fiber. *Nat Struct Mol Biol*, **16**, 534-540.
61. Cui, Y. and Bustamante, C. (2000) Pulling a single chromatin fiber reveals the forces that maintain its higher-order structure. *Proceedings of the National Academy of Sciences*, **97**, 127-132.
62. Recouvreux, P., Lavelle, C., Barbi, M., Conde e Silva, N., Le Cam, E., Victor, J.-M. and Viovy, J.-L. (2011) Linker Histones Incorporation Maintains Chromatin Fiber Plasticity. *Biophysical Journal*, **100**, 2726-2735.
